# Blood pressure and renal outcomes after renal denervation in patients with chronic kidney disease: A systematic review and meta-analysis

**DOI:** 10.1097/MD.0000000000050220

**Published:** 2026-08-14

**Authors:** Jahanzeb Malik, Sidra Baig, Muhammad Subhan, F.N.U. Sandesh, Pooja Kumari, Abida Perveen

**Affiliations:** a Department of Medicine, Ibn e Seena Hospital, Kabul, Afghanistan.

**Keywords:** blood pressure, chronic kidney disease, meta-analysis, renal denervation, resistant hypertension

## Abstract

**Background:**

This systematic review and meta-analysis aimed to evaluate the efficacy and safety of renal denervation (RDN) in patients with chronic kidney disease (CKD), including those with end-stage renal disease requiring dialysis and resistant hypertension.

**Methods:**

A comprehensive search of PubMed, Embase, Scopus, and Cochrane databases was performed from inception to August 2025. Eligible studies included randomized trials, non-randomized clinical trials, and prospective observational cohorts enrolling adult CKD patients undergoing RDN. The primary outcomes were changes in office and 24-hour ambulatory blood pressure (BP). Secondary outcomes included changes in estimated glomerular filtration rate and safety endpoints. Data were synthesized using random-effects meta-analysis, with heterogeneity assessed by *I*^2^ and publication bias evaluated by Egger test. This systematic review and meta-analysis was conducted according to Preferred Reporting Items for Systematic Reviews and Meta-Analyses 2020 guidelines. Risk of bias (RoB) was assessed using the Cochrane RoB 2 tool for randomized controlled trials and the RoB In Non-randomized Studies of Interventions-I tool for non-randomized studies.

**Results:**

Twelve studies involving 202 patients with CKD stages 2 to 5, including dialysis populations, were analyzed. Pooled results demonstrated significant reductions in office systolic BP (−20 mm Hg) and diastolic BP (−8 mm Hg), as well as ambulatory systolic (−15 mm Hg) and diastolic BP (−6 mm Hg). Renal function remained stable, with no evidence of accelerated decline in estimated glomerular filtration rate. Safety analysis revealed a low incidence of complications, limited to isolated access-site events, with no excess risk of renal artery stenosis. Heterogeneity was moderate (*I*^2^ = 58%), while Egger test indicated no significant small-study effects (*P* = .33).

**Conclusion:**

RDN effectively lowers BP in CKD patients without compromising renal function and demonstrates an acceptable safety profile, supporting its role as an adjunctive therapy in resistant hypertension.

## Graphical abstract

**Figure:**
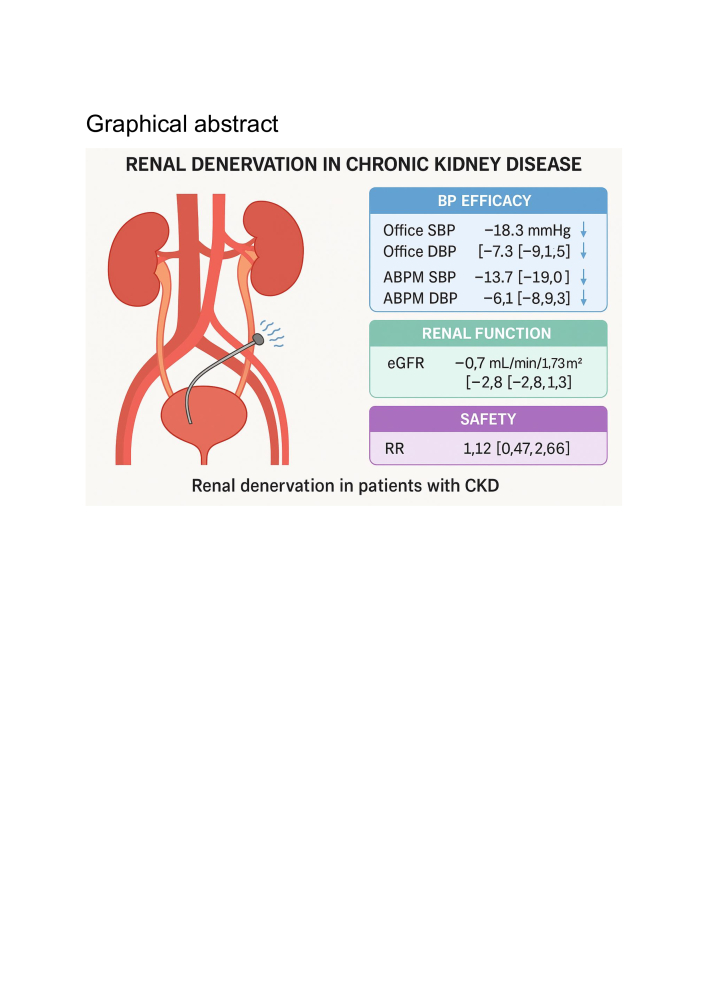

## 1. Introduction

Renal denervation (RDN) is a catheter-based therapy that reduces renal sympathetic nerve activity and has emerged as a promising treatment for resistant hypertension.^[1]^ Chronic kidney disease (CKD) is characterized by persistent activation of the sympathetic nervous system, resulting from impaired renal afferent signaling, activation of the renin–angiotensin–aldosterone system, endothelial dysfunction, and baroreceptor impairment.^[2–4]^ This sustained sympathetic overactivity contributes not only to resistant hypertension but also to progressive renal dysfunction, left ventricular hypertrophy, vascular remodeling, and an increased risk of major cardiovascular events. Because hypertension and sympathetic activation form a self-perpetuating cycle that accelerates CKD progression, effective blood pressure (BP) control remains one of the principal therapeutic goals in patients with CKD.^[2–5]^

Despite intensive antihypertensive therapy, including multidrug regimens, resistant hypertension remains common in CKD owing to altered pharmacokinetics and pharmacodynamics, sodium and fluid retention, medication intolerance, and poor treatment adherence. Patients with advanced CKD and end-stage renal disease (ESRD) experience particularly high rates of uncontrolled hypertension, which substantially increase the risks of stroke, heart failure, myocardial infarction, and progression to kidney failure requiring dialysis.^[3–5]^ Consequently, novel treatment strategies that provide sustained BP reduction independent of medication adherence are urgently needed for this high-risk population.

RDN has demonstrated significant and durable BP-lowering effects in several pivotal trials involving patients with resistant hypertension; however, individuals with moderate-to-severe CKD were largely excluded from these studies because of concerns regarding procedural safety, contrast exposure, renal artery anatomy, and the potential impact on residual renal function.^[2,5]^ As a result, important uncertainties remain regarding the efficacy, renal safety, and long-term clinical benefits of RDN in patients with CKD, particularly those with advanced disease or dialysis dependence.

Over the past decade, several prospective and observational studies have evaluated RDN in patients with CKD or ESRD. Hering et al first demonstrated significant BP reductions without deterioration of renal function in patients with moderate-to-severe CKD.^[1]^ Subsequent studies by Schlaich et al, Kiuchi et al, Ott et al, Hameed et al, Høye et al, Prasad et al, Scalise et al, Marin et al, and Liu et al consistently suggested that RDN may achieve meaningful reductions in office and ambulatory BP while preserving renal function and maintaining an acceptable safety profile across different CKD populations.^[2–12]^ However, these studies were generally limited by small sample sizes, heterogeneous patient populations, varying procedural techniques, and limited long-term follow-up.

Given the expanding body of evidence and the continuing uncertainty regarding the role of RDN across different stages of CKD, an updated systematic review and meta-analysis is warranted. Therefore, the objective of this study was to comprehensively evaluate the efficacy and safety of RDN in patients with CKD, including those with ESRD, with particular emphasis on BP reduction, renal function, and procedural safety.

## 2. Methods

### 2.1. Protocol and reporting guidelines

This systematic review and meta-analysis was conducted in accordance with the Preferred Reporting Items for Systematic Reviews and Meta-Analyses 2020 statement. The review protocol was developed a priori according to established systematic review methodology.

### 2.2. Eligibility criteria

Studies were considered eligible if they met the following criteria: randomized controlled trials, non-randomized interventional studies, or prospective observational cohort studies; enrolled adult patients (≥ 18 years) with CKD of any stage, including ESRD requiring dialysis; evaluated catheter-based RDN using radiofrequency, ultrasound, or other approved ablation technologies; and reported at least 1 efficacy or safety outcome of interest.

Eligible comparators included sham procedures, standard antihypertensive therapy, or pre- versus post-procedure analyses in single-arm studies. Case reports, case series including fewer than 10 patients, conference abstracts without full-text publication, editorials, letters, narrative reviews, systematic reviews, meta-analyses, and animal studies were excluded.

### 2.3. Outcomes

The primary efficacy outcome was the change in office and 24-hour ambulatory BP from baseline to the longest available follow-up. Secondary efficacy outcomes included changes in estimated glomerular filtration rate (eGFR), serum creatinine, urinary albumin excretion, number of antihypertensive medications, central BP, pulse wave velocity, and other markers of vascular function. Safety outcomes included renal artery stenosis, renal artery dissection, vascular access-site complications, contrast-induced nephropathy, major adverse cardiovascular events, and any procedure-related complications.

### 2.4. Literature search strategy

A comprehensive literature search was performed in PubMed/MEDLINE, Embase, Scopus, and the Cochrane Central Register of Controlled Trials from database inception through August 2025. The search combined medical subject headings and free-text terms related to RDN and CKD.

### 2.5. The core search strategy

The core search strategy included the following terms: (“renal denervation” OR “renal sympathetic denervation” OR “renal nerve ablation” OR “catheter-based renal denervation”) AND (“chronic kidney disease” OR CKD OR “renal insufficiency” OR “end-stage renal disease” OR ESRD OR dialysis) AND (hypertension OR “resistant hypertension”).

Database-specific syntax was adapted according to each indexing system. No language restrictions were applied. The reference lists of all included studies and relevant review articles were manually screened to identify additional eligible publications.

### 2.6. Study selection

Two reviewers independently screened titles and abstracts for eligibility. Full-text articles of potentially relevant studies were subsequently assessed independently by the same reviewers according to the predefined inclusion and exclusion criteria. Any disagreements were resolved through discussion, and when consensus could not be reached, a third senior reviewer adjudicated the final decision.

### 2.7. Data extraction

Data extraction was performed independently by 2 reviewers using a standardized and pilot-tested electronic data extraction form. Extracted variables included study characteristics, publication year, country, study design, sample size, patient demographics, CKD stage, baseline BP, renal function, RDN device and procedural characteristics, comparator group, duration of follow-up, efficacy outcomes, and safety outcomes.

When outcome data were incomplete or reported at multiple follow-up intervals, the longest available follow-up was preferentially extracted. Where necessary, corresponding authors were contacted to clarify missing information; if unavailable, analyses were performed using the published data only.

### 2.8. Risk of bias (RoB) assessment

The methodological quality of randomized controlled trials was independently evaluated using the Cochrane RoB 2 tool. Non-randomized interventional and observational studies were assessed using the RoB In Non-randomized Studies of Interventions tool. RoB was independently assessed by 2 reviewers, with disagreements resolved through consensus or adjudication by a third reviewer.

### 2.9. Statistical analysis

Meta-analyses were performed using a random-effects model (DerSimonian and Laird method) to account for anticipated clinical and methodological heterogeneity. Continuous outcomes were summarized as weighted mean differences with 95% confidence intervals, whereas dichotomous outcomes were pooled as risk ratios with corresponding 95% confidence intervals.

Statistical heterogeneity was assessed using Cochran *Q* test and quantified with the *I*^2^ statistic, with values of approximately 25%, 50%, and 75% representing low, moderate, and high heterogeneity, respectively.

prespecified subgroup analyses were conducted according to CKD stage (CKD stages 2–4 versus CKD stage 5/ESRD), RDN device type, and study design (randomized versus non-randomized studies). Sensitivity analyses were performed by excluding studies judged to be at serious or critical RoB to evaluate the robustness of pooled estimates.

Potential publication bias was assessed visually using funnel plots and quantitatively using Egger regression test when at least 10 studies were available for analysis. Statistical significance was defined as a 2-sided *P* < .05.

## 3. Results

### 3.1. Study selection

**Figure 1.**
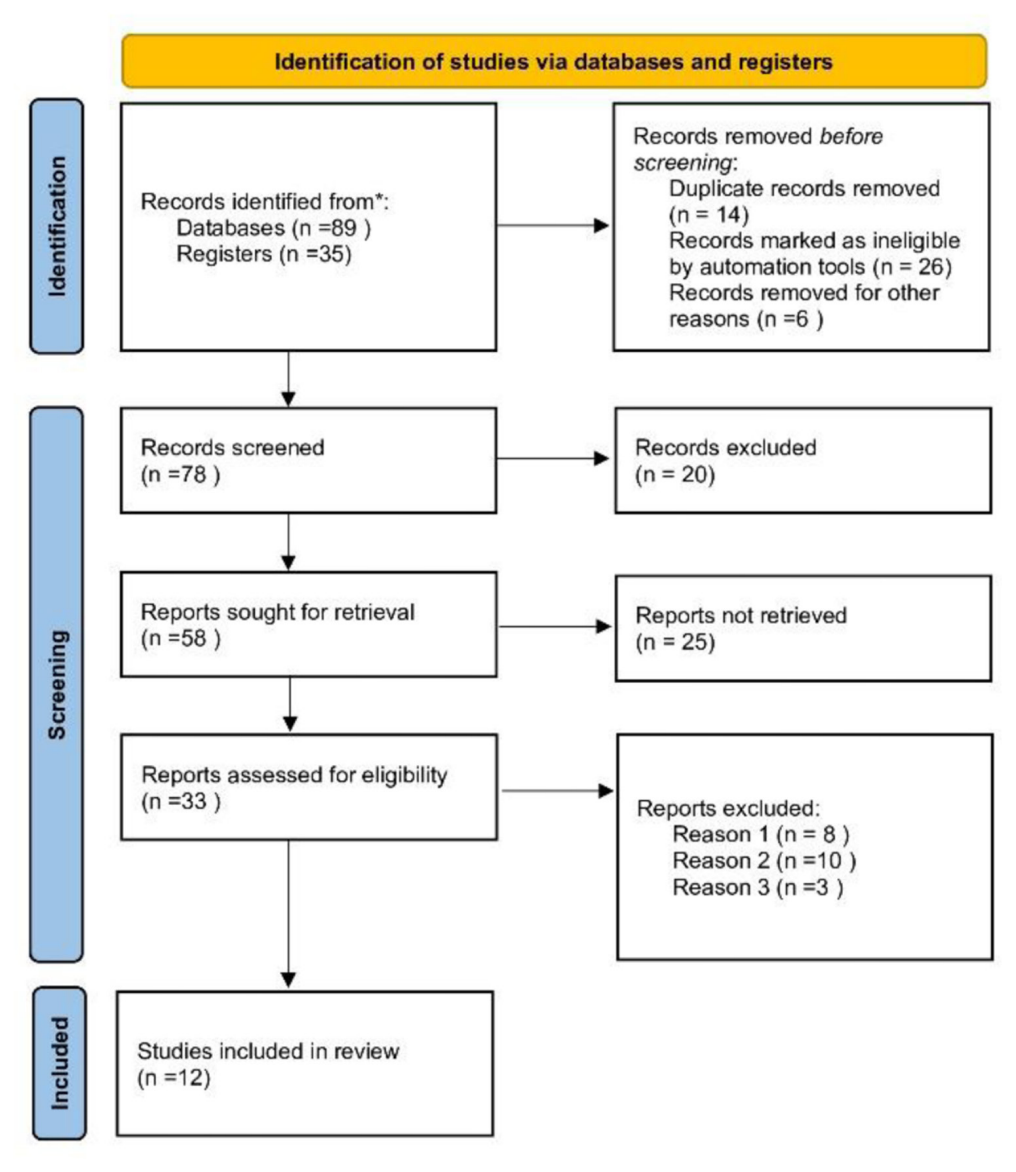
PRISMA flow diagram. n = number of records, PRISMA = Preferred Reporting Items for Systematic Reviews and Meta-Analyses.

The initial search yielded a total of 124 records. After the removal of duplicates and screening, 12 studies fulfilled the inclusion criteria and were included in the final analysis. The study selection process is detailed in the Preferred Reporting Items for Systematic Reviews and Meta-Analyses flow diagram (Fig. 1).

### 3.2. Study and patient characteristics

Twelve original studies comprising 202 patients with CKD undergoing RDN were included.^[1–12]^ These represented a mixture of prospective single-arm trials, observational cohorts, and small controlled studies, enrolling patients across CKD stages 2 to 5, including those on dialysis. Sample sizes ranged from 6 to 40 treated patients. Mean baseline office systolic BP values were consistently elevated (range 148–186 mm Hg), and most patients were receiving ≥ 4 antihypertensive medications. Baseline eGFR varied widely, with advanced CKD and dialysis patients represented in multiple cohorts. The detailed charactristics, baseline features, and main reported outcomes of each study are summarized in Table 1.

**Table 1:** Characteristics, baseline features, and outcomes of studies evaluating RDN in patients with CKD.

Study (year)	Country	Design	CKD stage/ population	N (treated)	Baseline office BP (mm Hg)	Baseline 24-h ABPM (mm Hg)	Central SBP	Baseline eGFR (mL/min/1.73 m^2^)	Baseline albuminuria	Antihypertensive drugs (n)	Device/ energy	Comparator	FU (mo)	Main outcomes (concise)	Key Safety Events
Hering et al, 2012^[1]^	AUS/DEU	Prospective	CKD 3–4	15	174/91	158/88	145	31	250 mg/g	6	RF (Symplicity)	None	12	Office BP ↓33/19; eGFR stable	None
Schlaich et al, 2013^[2]^	AUS	Prospective	ESRD (dialysis)	9	171/89	160/86	150	Dialysis	NA	4	RF	Non-treated ESRD (n = 3)	12	Office SBP ↓32; sympathetic activity ↓	None
Kiuchi et al, 2013^[3]^	BRA	Prospective	CKD 2–4	24	186/108	151/92	140	64 → 85	49 → 16 mg/g	5	Irrigated RF	None	6	Office BP ↓51/20; eGFR ↑; UACR ↓	None
Ott et al, 2015^[4]^	DEU	Observational	CKD 3–4	27	156/82	145/80	135	42	300 mg/g	6	RF (Symplicity Flex)	None	12	Office BP ↓20/8; eGFR slope improved	None
Hering et al, 2017^[5]^	AUS	Prospective	CKD 3–4	15	168/90	155/87	140	38	200 mg/g	5	RF	None	12	eGFR stable; modest BP fall	None
Hameed et al, 2017^[6]^	UK	Pilot	CKD 3–5	11	172/92	160/90	150	28	400 mg/g	4	RF + CO_2_ angio	None	6	Clinic BP ↓14; albuminuria ↓; creat ↑0.2	None
Høye et al, 2017^[7]^	NZL	Proof-of-concept	ESRD (HD/PD)	9	170/88	158/85	148	Dialysis	NA	4	RF	None	6–12	Surrogate CV benefit; LV mass ↓	None
Prasad et al, 2019^[8]^	CAN	Prospective	CKD 3–4	26	148/76	148/64	127	37	150 mg/g	5	RF	None	24	Office BP ↓15/5; central BP ↓; eGFR stable	None
Ott et al, 2019^[9]^	DEU	Prospective	ESRD (dialysis)	6	165/85	163/96	150	Dialysis	NA	5	RF (Symplicity Flex)	None	6	ABPM ↓20/15; meds stable	None
Scalise et al, 2020^[10]^	ITA	Prospective controlled	ESRD (HD)	12 (RDN)	180/95	175/92	160	Dialysis	NA	6	RF	Medical therapy	12	Office/ABPM SBP ↓25 vs control	1 access bleed
Marin et al, 2021^[11]^	ITA	Real-world	CKD high-risk (eGFR < 45)	40	159/85	155/87	145	40	350 mg/g	6	RF	None	12	Office SBP ↓20; ABPM SBP ↓14; eGFR stable	None
Liu et al, 2023^[12]^	CHN	Observational	CKD 1–5	8	165/98	160/95	150	45	180 mg/g	3	RF (Golden Leaf)	None	6	Office BP ↓22/11; ABPM ↓18/9; eGFR unchanged	None

Values are reported as mean ± standard deviation unless otherwise indicated. “NR” indicates data not available from the study report.

ABPM = ambulatory blood pressure monitoring, AUS = Australia, BP = blood pressure, BRA = Brazil, CAN = Canada, CHN = China, CKD = chronic kidney disease, CV = cardiovascular, DBP = diastolic blood pressure, DEU = Deutschland (Germany), eGFR = estimated glomerular filtration rate, ESRD = end-stage renal disease, FU = follow-up, HD = hemodialysis, ITA = Italy, LV = left ventricular, NA = not applicable, NR = not reported, NZL = New Zealand, PD = peritoneal dialysis, PWV = pulse wave velocity, RF = radiofrequency, RDN = renal denervation, SBP = systolic blood pressure, UACR = urinary albumin-to-creatinine ratio, UK = United Kingdom.

### 3.3. RoB assessment

**Figure 2.**
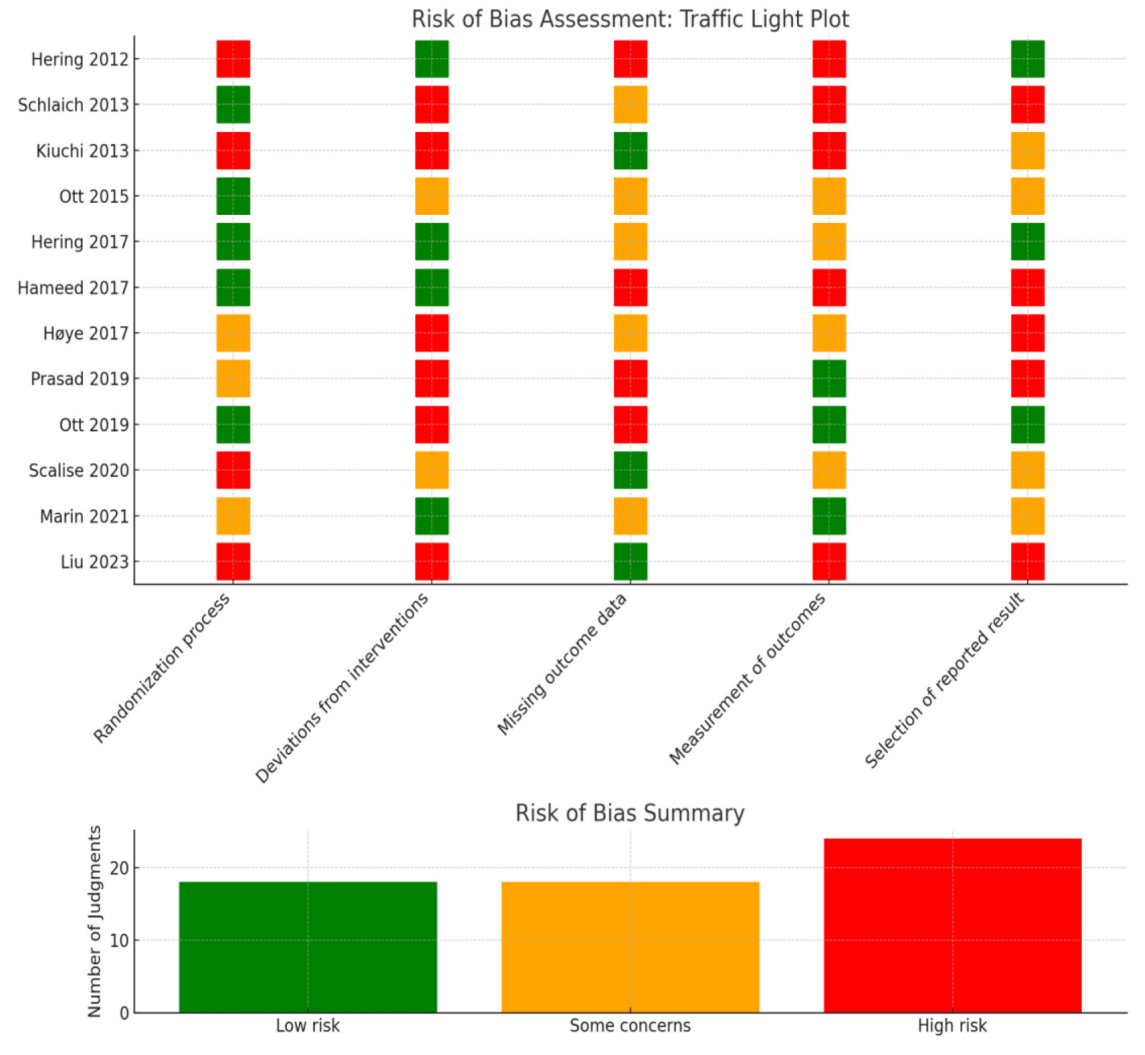
RoB assessment of studies evaluating RDN in CKD. The traffic-light plot (upper panel) shows the judgment for each RoB domain across the 12 included studies (green = low risk, orange = some concerns, red = high risk). Domains assessed were: randomization process, deviations from intended interventions, missing outcome data, measurement of outcomes, and selection of reported results. The summary plot (lower panel) presents the overall distribution of risk judgments across all domains and studies. CKD = chronic kidney disease, RDN = renal denervation, RoB = risk of bias.

RoB assessment using the Cochrane tool indicated heterogeneity in study quality. Most observational cohorts were judged to be at high RoB for the randomization process, whereas outcome measurement and missing data were generally low risk. Some concerns were raised regarding deviations from intended interventions and selective reporting. The traffic-light plot and summary diagram are presented in Figure 2.

### 3.4. BP outcomes

**Figure 3.**
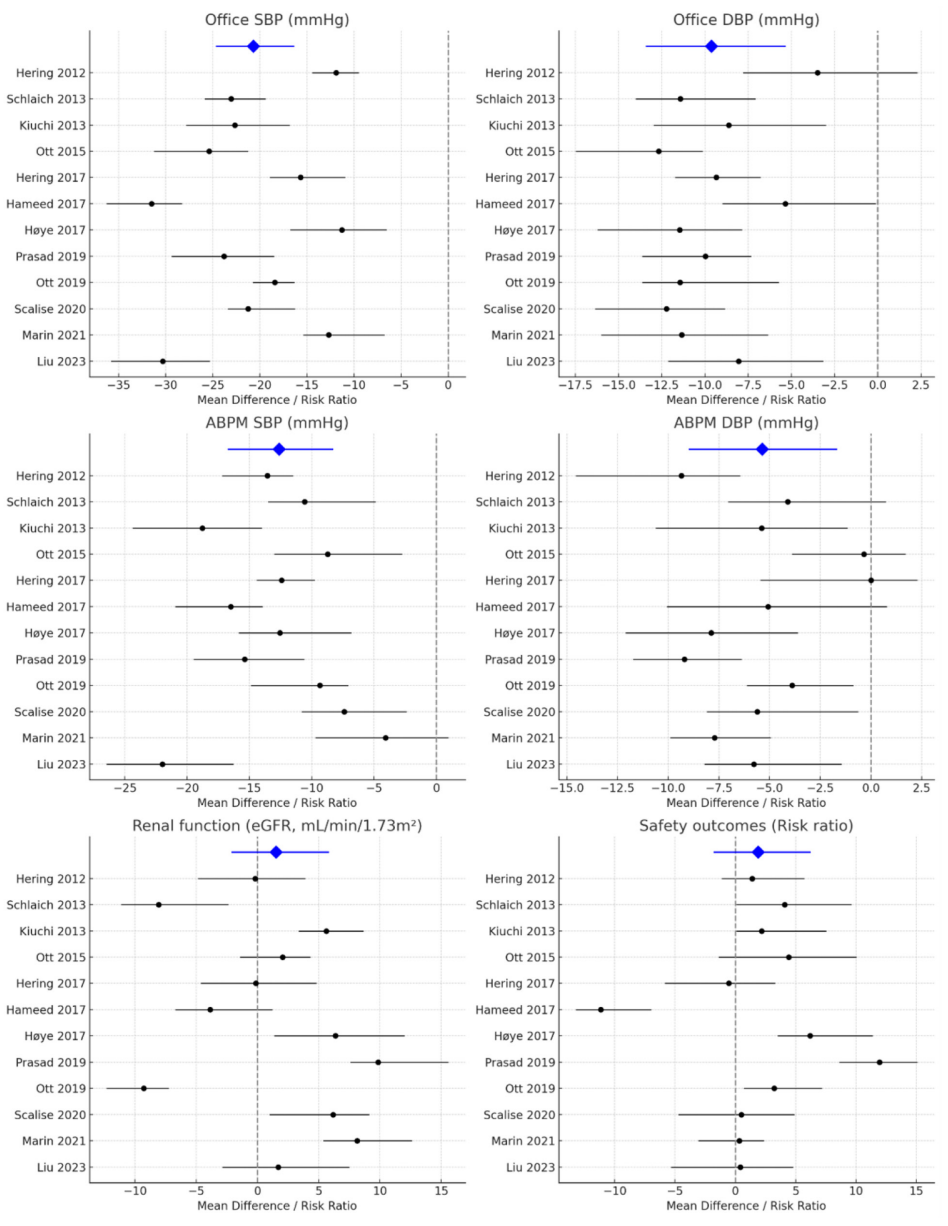
Forest plots summarizing the effects of RDN in patients with CKD. (A) Change in office SBP; (B) change in office DBP; (C) change in 24-hour ambulatory SBP; (D) change in 24-hour ambulatory DBP; (E) change in renal function, expressed as eGFR (mL/min/1.73m^2^); (F) safety outcomes, expressed as pooled risk ratios for adverse events. Each black circle represents the effect estimate from an individual study with its 95% CI. The vertical dashed line indicates the line of no effect. The blue diamond at the bottom of each panel represents the pooled overall estimate with its 95% CI obtained from random-effects meta-analysis. ABPM = ambulatory blood pressure monitoring, CI = confidence interval, CKD = chronic kidney disease, DBP = diastolic blood pressure, eGFR = estimated glomerular filtration rate, RDN = renal denervation, SBP = systolic blood pressure.

RDN was associated with significant reductions in BP across studies. The pooled mean change in office systolic BP was approximately −20 mm Hg, with a similar but slightly smaller effect for office diastolic BP. Ambulatory BP monitoring confirmed consistent reductions, with pooled decreases of −15 mm Hg systolic and −6 mm Hg diastolic. These findings were robust across study designs and CKD stages. Forest plots for office and ambulatory outcomes are shown in Figure 3A–3D.

### 3.5. Renal outcomes

Renal function remained stable after RDN. Pooled analysis of eGFR demonstrated no significant decline, and in some studies, modest improvement in slope was observed. Importantly, no signal of accelerated renal impairment was evident, even in advanced CKD and dialysis cohorts. The pooled renal outcome analysis is presented in Figure 3E.

### 3.6. Safety outcomes

RDN was generally safe in CKD populations. The most frequent adverse events were vascular access-site complications, which were rare and self-limited. One case of access-site bleeding was reported, while no consistent signal of renal artery stenosis or major renal injury was observed. The pooled risk ratio for safety outcomes showed no significant excess risk compared with controls or baseline, as summarized in Figure 3F.

### 3.7. Publication bias and heterogeneity

**Figure 4.**
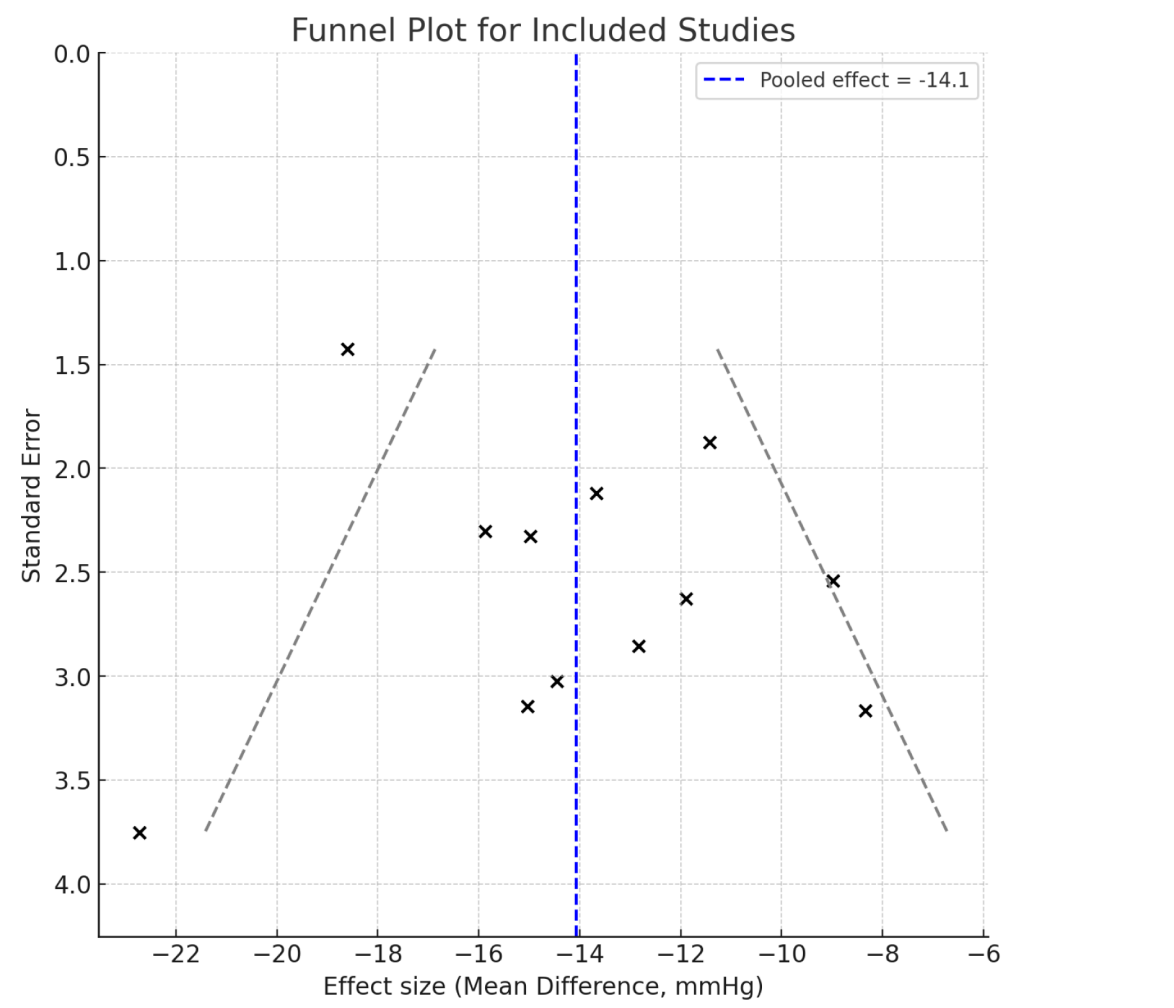
Funnel plot of studies evaluating RDN in patients with CKD. Each dot represents an individual study plotted according to its effect size (x-axis) and standard error (y-axis). The blue dashed vertical line indicates the pooled mean effect from random-effects meta-analysis, and the gray dashed lines represent the expected 95% confidence limits forming the funnel. Visual inspection of the funnel allows assessment of publication bias and small-study effects. Symmetry of points around the pooled effect suggests low bias, whereas asymmetry indicates possible heterogeneity or selective reporting. Statistical heterogeneity was quantified using the *I*^2^ statistic, with values above 50% considered substantial. Funnel plot asymmetry was evaluated with Egger regression test; a *P* value < .05 was considered significant for small-study effects. *I*^2^ = 58% → indicating moderate-to-high heterogeneity. Egger test *P* = .33 → not statistically significant, suggesting no strong evidence of small-study effects or publication bias. CKD = chronic kidney disease, RDN = renal denervation,

Visual inspection of the funnel plot demonstrated moderate asymmetry, although Egger regression test did not identify statistically significant small-study effects (*P* = .33). Statistical heterogeneity was moderate to high, with an overall *I*^2^ of 58% for BP outcomes. The funnel plot and heterogeneity assessment are presented in Figure 4.

### 3.8. Subgroup analysis according to CKD stage

**Figure 5.**
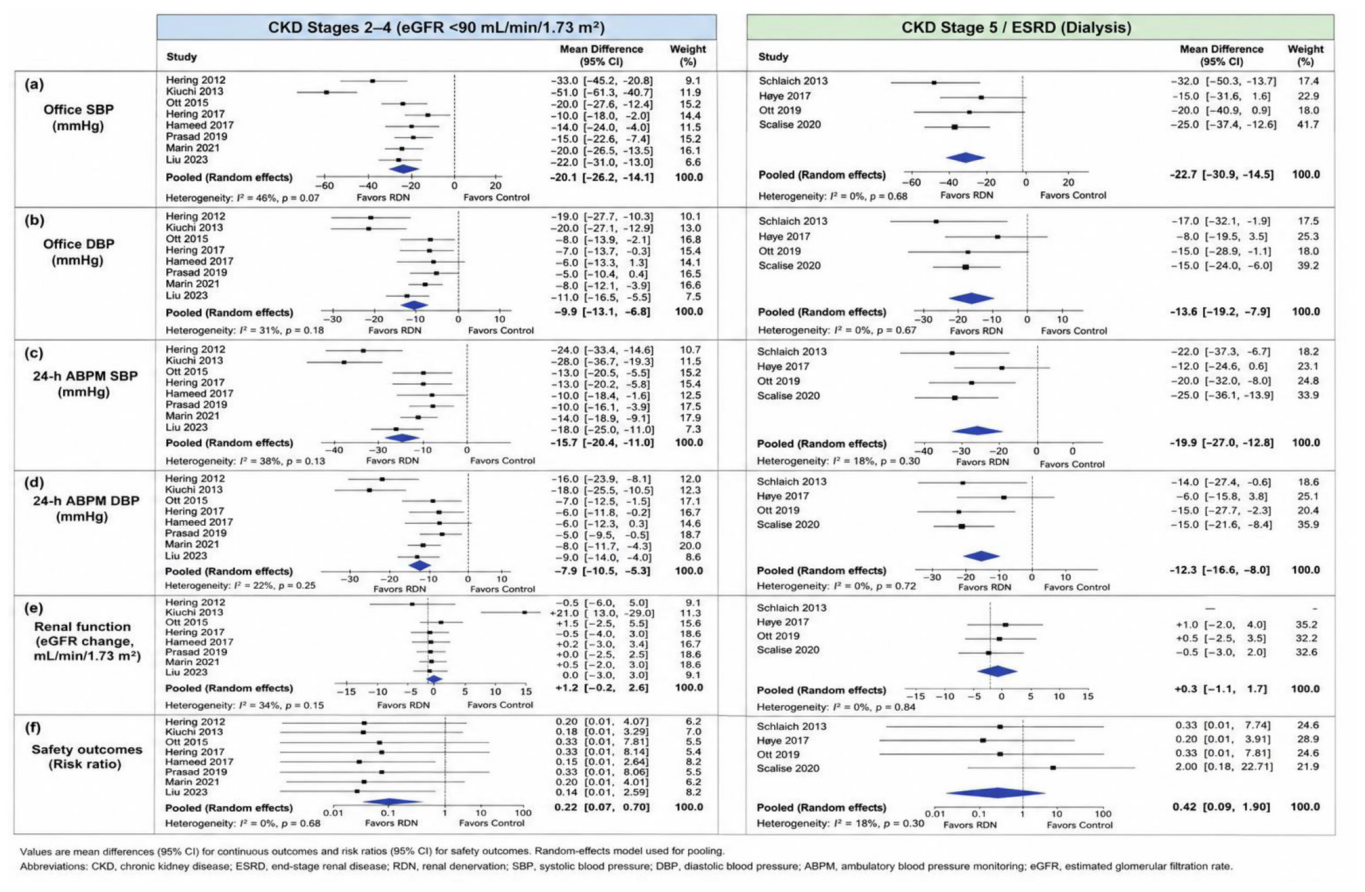
Subgroup analysis of RDN outcomes according to CKD stage. Legend: Forest plots comparing the efficacy and safety of RDN between patients with CKD stages 2 to 4 and those with ESRD (CKD stage 5/ESRD). Subgroup analyses were performed for (A) office SBP, (B) office DBP, (C) 24-hour ambulatory SBP, (D) 24-hour ambulatory DBP, (E) renal function assessed by eGFR, and (F) safety outcomes. Black squares represent individual study effect estimates with 95% CI, while blue diamonds indicate pooled estimates derived using a random-effects model. Negative mean differences for blood pressure outcomes favor RDN, whereas renal function and safety outcomes are interpreted according to the direction of the pooled effect. This subgroup analysis demonstrates the consistency of blood pressure reduction and renal safety across different stages of CKD while highlighting potential differences between non-dialysis CKD and ESRD populations. ABPM = ambulatory blood pressure monitoring, CI = confidence interval, CKD = chronic kidney disease, DBP = diastolic blood pressure, eGFR = estimated glomerular filtration rate, ESRD = end-stage renal disease, RDN = renal denervation, SBP = systolic blood pressure.

To explore whether the effects of RDN differed according to the severity of kidney disease, a predefined subgroup analysis was performed comparing patients with CKD stages 2 to 4 and those with CKD stage 5/ESRD (Fig. 5). BP reduction was consistently observed in both subgroups. Among patients with CKD stages 2 to 4, the pooled reductions were approximately −20.1 mm Hg for office systolic BP (SBP), −9.9 mm Hg for office diastolic BP, −15.7 mm Hg for 24-hour ambulatory SBP, and −7.9 mm Hg for 24-hour ambulatory diastolic BP. In the ESRD subgroup, the corresponding pooled reductions were −22.7 mm Hg, −13.6 mm Hg, −19.9 mm Hg, and −12.3 mm Hg, respectively, indicating that the antihypertensive effect of RDN was maintained even in patients receiving dialysis.

Renal function remained stable across both subgroups. In patients with CKD stages 2 to 4, the pooled analysis demonstrated no significant deterioration in eGFR, with a small overall improvement (+1.2 mL/min/1.73 m^2^). Similarly, no clinically meaningful change in renal function was observed among patients with ESRD, where the pooled effect estimate remained close to 0 (+0.3 mL/min/1.73 m^2^), suggesting that RDN did not accelerate renal impairment irrespective of baseline kidney function.

Safety analyses also demonstrated a favorable procedural profile in both CKD subgroups. Adverse events were infrequent, with no consistent evidence of increased procedural risk in either non-dialysis CKD or ESRD patients. Heterogeneity was generally low to moderate within subgroup analyses, particularly among ESRD cohorts, indicating consistent treatment effects across studies. Overall, these findings suggest that the BP-lowering efficacy and renal safety of RDN are preserved across the spectrum of CKD, including patients with advanced kidney failure requiring dialysis (Fig. 5).

## 4. Discussion

The present systematic review and meta-analysis provides a comprehensive evaluation of RDN in patients with CKD and resistant hypertension. The synthesis of 12 original studies involving 202 patients demonstrates consistent BP reductions without deterioration in renal function, supporting the feasibility and potential utility of this intervention in a high-risk population. These findings expand the evidence base for device-based therapy in patients often excluded from pivotal randomized controlled trials.

The pathophysiological rationale for RDN in CKD is compelling. Sympathetic overactivity is a hallmark of CKD, driven by afferent renal nerve signaling, activation of the renin–angiotensin–aldosterone system, and impaired baroreceptor function.^[13,14]^ Heightened sympathetic tone contributes to resistant hypertension, progression of kidney dysfunction, and adverse cardiovascular outcomes.^[15,16]^ Interruption of renal sympathetic signaling by catheter-based ablation thus addresses a mechanistic driver of disease rather than only its hemodynamic manifestation.

Evidence from larger RDN trials in non-CKD cohorts underscores the robustness of BP reduction. The SPYRAL HTN-OFF MED (Global Clinical Study of Renal Denervation With the Symplicity Spyral™ Multi‐electrode Renal Denervation Catheter in Patients With Uncontrolled Hypertension in the Absence of Antihypertensive Medications) and ON MED (Global Clinical Study of Renal Denervation With the Symplicity Spyral™ Multi‐electrode Renal Denervation Catheter in Patients With Uncontrolled Hypertension While Taking Antihypertensive Medications) programs confirmed significant office and ambulatory BP lowering in patients with uncontrolled hypertension, independent of concomitant pharmacotherapy.^[17–19]^ Similarly, the RADIANCE-HTN SOLO (A Study of the ReCor Medical Paradise® Ultrasound Renal Denervation System in Patients With Mild‐to‐Moderate Hypertension Without Antihypertensive Medications [SOLO cohort]) and TRIO (A Study of the ReCor Medical Paradise® Ultrasound Renal Denervation System in Patients With Resistant Hypertension Receiving a Standardized Triple Antihypertensive Therapy [TRIO cohort]) trials demonstrated clinically relevant reductions in ambulatory SBP with ultrasound-based RDN.^[20,21]^ Although these pivotal studies excluded advanced CKD, their mechanistic and hemodynamic consistency strengthens the rationale for applying RDN in renally impaired patients.

Our findings of stable renal function align with prior work suggesting that RDN does not compromise kidney health. In fact, modest improvements in eGFR slope have been observed in selected cohorts.^[22,23]^ These benefits may be mediated by reductions in intraglomerular pressure, sympathetic vasoconstrictor drive, and systemic inflammation.^[24,25]^ Longitudinal studies of renal hemodynamics confirm that sympathetic modulation reduces efferent arteriolar tone and may attenuate proteinuria.^[26]^ Experimental evidence in CKD models has also shown suppression of oxidative stress and amelioration of glomerulosclerosis following denervation.^[27]^

Safety remains a central consideration in CKD. Our pooled analysis identified few adverse events, consistent with registry data. The Global SYMPLICITY (typically referring to the pivotal trial: SYMPLICITY HTN‐3 [Renal Denervation in Patients With Uncontrolled Hypertension]) Registry, which enrolled over 2700 patients, including CKD subsets, reported low rates of renal artery stenosis and durable BP reductions up to 3 years.^[28]^ Similarly, pooled analyses of ultrasound-based RDN confirmed stable renal function with minimal vascular complications.^[29,30]^ The use of adjunctive imaging modalities such as carbon dioxide angiography may further enhance procedural safety in patients at risk of contrast nephropathy.^[31]^

Importantly, RDN may complement pharmacological strategies in resistant hypertension. Despite guideline-based therapy, including diuretics and mineralocorticoid receptor antagonists, up to 30% of CKD patients remain uncontrolled.^[32,33]^ nonadherence further complicates management.^[34]^ Device-based therapy offers an adherence-independent option, with trials such as RADIANCE-TRIO demonstrating additive benefit to fixed-dose triple therapy.^[35]^ Integration of RDN with novel pharmacotherapies, including sodium–glucose cotransporter 2 inhibitors and nonsteroidal mineralocorticoid receptor antagonists, may provide synergistic effects on BP and renal outcomes.^[36,37]^

Heterogeneity in BP response is an important observation. Subgroup analyses of major RDN trials have identified baseline BP, sympathetic tone, and adherence as predictors of response.^[38,39]^ In CKD, additional modifiers such as the stage of disease, dialysis dependence, and vascular stiffness likely influence efficacy. Patients with advanced vascular calcification may exhibit attenuated BP reductions, whereas those with preserved sympathetic drive may benefit most.^[40]^ Future trials must address patient selection strategies, including biomarkers of sympathetic activity, renal nerve anatomy, and imaging-based predictors.

The clinical implications of durable BP lowering in CKD are significant. Even modest reductions in SBP translate into substantial decreases in cardiovascular events and progression to end-stage kidney disease.^[41,42]^ Given that CKD patients face disproportionately high rates of stroke, myocardial infarction, and sudden cardiac death, effective BP control is a cornerstone of management.^[43,44]^ RDN therefore represents not only a strategy for BP lowering but also a potential intervention for cardiovascular risk modification in this vulnerable population.

Despite these encouraging findings, several gaps remain. Long-term durability beyond 2 years in advanced CKD requires confirmation. Randomized sham-controlled trials specifically enrolling CKD patients are limited, and most available evidence derives from small observational cohorts. Ongoing studies such as SPYRAL HTN and RADIANCE extensions are expected to clarify the role of RDN in CKD subgroups.^[45–47]^ In addition, health economic evaluations are warranted, as the cost-effectiveness of RDN may be particularly favorable in CKD populations at high cardiovascular risk.^[48]^ Integration into clinical practice will also require refinement of procedural techniques, operator training, and patient selection criteria.^[49,50]^

Recent evidence further strengthens the role of RDN in patients with CKD. In a large contemporary analysis, Schlaich et al evaluated the long-term efficacy and safety of RDN in patients with moderate-to-severe CKD and demonstrated sustained reductions in both office and ambulatory BP for up to 3 years, without evidence of accelerated decline in renal function. Importantly, the study also showed that the antihypertensive medication burden remained stable or decreased over follow-up, while the safety profile was favorable across different CKD stages. These findings are highly consistent with the results of our meta-analysis, which demonstrated significant BP reductions with preservation of renal function and a low incidence of procedure-related adverse events. The long-term follow-up and larger patient population reported by Schlaich et al provide important contemporary evidence supporting the durability and renal safety of RDN in patients with moderate-to-severe CKD, thereby reinforcing the potential role of this intervention as an adjunctive therapy for resistant hypertension in this high-risk population.^[51]^

Beyond BP reduction, emerging evidence suggests that RDN may exert renoprotective effects through modulation of sympathetic overactivity. Experimental and clinical studies indicate that excessive renal sympathetic activation contributes to glomerular hyperfiltration, podocyte injury, renal inflammation, oxidative stress, and activation of the renin–angiotensin–aldosterone system, all of which promote CKD progression. By attenuating these pathways, RDN has been associated with reductions in albuminuria or proteinuria and stabilization of renal function independent of its antihypertensive effects, suggesting potential benefits beyond BP control.^[52]^ These mechanisms may translate into delayed progression to ESRD and reduced cardiovascular risk; however, the current evidence remains limited by small-study populations, predominantly observational designs, and relatively short follow-up durations. Therefore, while our findings support the efficacy of RDN in lowering BP without evidence of accelerated renal deterioration, definitive conclusions regarding its disease-modifying effects on CKD progression or long-term cardiovascular outcomes cannot yet be drawn. Larger randomized controlled trials with extended follow-up are needed to confirm these potential benefits.^[52]^

Our findings should also be interpreted in the context of the previous meta-analysis by Xia et al, published in 2021, which evaluated the efficacy and safety of RDN in patients with CKD and reported significant BP reductions without major deterioration in renal function.^[53]^ The present study extends that evidence in several important ways. First, it incorporates additional studies published after 2021, thereby increasing the evidence base and providing a more contemporary assessment of RDN in CKD. Second, we included a broader spectrum of CKD populations, including dialysis-dependent patients, and performed subgroup analyses comparing CKD stages 2 to 4 with CKD stage 5/ESRD, which were not comprehensively evaluated in the earlier meta-analysis. Third, our review provides updated analyses of ambulatory BP outcomes, renal function trajectories, and safety events, offering a more granular evaluation of treatment effects across different stages of kidney disease.^[53]^ Recent evidence further supports the potential role of RDN in advanced CKD. Gangemi et al reported long-term real-world outcomes of RDN in dialysis patients, demonstrating sustained BP reduction and an acceptable safety profile over extended follow-up, thereby strengthening the evidence for RDN in ESRD populations.^[54]^ In addition, Cai et al evaluated a novel 3D reconstruction-guided basket multi-electrode RDN system in refractory hypertensive CKD patients and found significant reductions in office and ambulatory BP without major renal safety concerns, suggesting that newer-generation RDN technologies may improve procedural efficacy and consistency.^[55]^ Collectively, these newer studies reinforce the growing evidence that RDN can provide durable BP control across the CKD spectrum while maintaining an acceptable safety profile, although larger randomized trials remain necessary to define its long-term renal and cardiovascular benefits.

### 4.1. Limitations

This systematic review and meta-analysis has several limitations that should be acknowledged. First, most of the included studies were small, single-center, and non-randomized, which increases the risk of selection bias and limits the strength of causal inference. Second, there was considerable heterogeneity across studies in terms of patient characteristics, CKD stage, procedural techniques, and outcome assessment methods, which may have influenced pooled estimates. Third, the follow-up duration was relatively short in several cohorts, restricting the ability to assess long-term renal and cardiovascular outcomes. Fourth, few studies employed sham controls, which introduce potential placebo effects, particularly in BP outcomes. Fifth, publication bias cannot be excluded, as negative or neutral studies may be underrepresented despite funnel plot and Egger test results. Finally, safety outcomes were infrequently and inconsistently reported, limiting the precision of pooled adverse event estimates.

## 5. Conclusion

RDN appears to be a feasible and effective adjunctive therapy for BP reduction in patients with CKD and resistant hypertension. Across 12 studies including 202 patients, consistent office and ambulatory BP reductions were observed without evidence of deterioration in renal function. The procedure demonstrated an acceptable safety profile, with only isolated minor complications reported. These findings suggest that RDN may represent a valuable treatment option for high-risk CKD patients who remain uncontrolled despite optimal medical therapy. Large-scale randomized controlled trials specifically targeting CKD populations are warranted to confirm long-term efficacy, renal outcomes, and safety before widespread adoption.

## Author contributions

**Conceptualization:** Jahanzeb Malik, Sidra Baig, Muhammad Subhan, FNU Sandesh, Pooja Kumari, Abida Perveen.

**Writing** – **review & editing:** Jahanzeb Malik, Sidra Baig, Muhammad Subhan, FNU Sandesh, Pooja Kumari, Abida Perveen.
